# Intrinsically photosensitive retinal ganglion cell function in relation to age: A pupillometric study in humans with special reference to the age-related optic properties of the lens

**DOI:** 10.1186/1471-2415-12-4

**Published:** 2012-04-03

**Authors:** Kristina Herbst, Birgit Sander, Henrik Lund-Andersen, Adam Elias Broendsted, Line Kessel, Michael Stormly Hansen, Aki Kawasaki

**Affiliations:** 1 Department of Ophthalmology, Glostrup Hospital, Ndr. Ringvej 57, 2600, Glostrup, Copenhagen, Denmark; 2 Department of Neuro-ophthalmology, Hôpital Ophtalmique Jules Gonin, Avenue de France 15, Lausanne, 1004, Switzerland; 3 Department of Clinical Sciences Ophthalmology, University of Umea, S90185, Umea, Sweden

## Abstract

**Background:**

The activity of melanopsin containing intrinsically photosensitive ganglion retinal cells (ipRGC) can be assessed by a means of pupil responses to bright blue (appr.480 nm) light. Due to age related factors in the eye, particularly, structural changes of the lens, less light reaches retina. The aim of this study was to examine how age and in vivo measured lens transmission of blue light might affect pupil light responses, in particular, mediated by the ipRGC.

**Methods:**

Consensual pupil responses were explored in 44 healthy subjects aged between 26 and 68 years. A pupil response was recorded to a continuous 20 s light stimulus of 660 nm (red) or 470 nm (blue) both at 300 cd/m^2^ intensity (14.9 and 14.8 log photons/cm^2^/s, respectively). Additional recordings were performed using four 470 nm stimulus intensities of 3, 30, 100 and 300 cd/m^2^. The baseline pupil size was measured in darkness and results were adjusted for the baseline pupil and gender. The main outcome parameters were maximal and sustained pupil contraction amplitudes and the postillumination response assessed as area under the curve (AUC) over two time-windows: early (0–10 s after light termination) and late (10–30 s after light termination). Lens transmission was measured with an ocular fluorometer.

**Results:**

The sustained pupil contraction and the early poststimulus AUC correlated positively with age (*p* = 0.02, *p* = 0.0014, respectively) for the blue light stimulus condition only.

The maximal pupil contraction amplitude did not correlate to age either for bright blue or red light stimulus conditions.

Lens transmission decreased linearly with age (*p* < 0.0001). The pupil response was stable or increased with decreasing transmission, though only significantly for the early poststimulus AUC to 300 cd/m^2^ light (*p* = 0.02).

**Conclusions:**

Age did not reduce, but rather enhance pupil responses mediated by ipRGC. The age related decrease of blue light transmission led to similar results, however, the effect of age was greater on these pupil responses than that of the lens transmission. Thus there must be other age related factors such as lens scatter and/or adaptive processes influencing the ipRGC mediated pupil response enhancement observed with advancing age.

## Background

The intrinsically photosensitive retinal ganglion cells (ipRGCs) are a small subset of retinal neurons which mediate non-image forming (NIF), light-dependent functions such as the pupil light reflex and circadian photoentrainment of biologic rhythms (sleep-awake, behaviour and mood) to the environmental light–dark cycle [1-4]. Melanopsin is the photoactive element of the ipRGCs. While it shares a spectral sensitivity nearly similar to rods (a peak sensitivity at 497 nm for rods [5] and a peak of appr.480 nm for melanopsin [3,4,6] in humans), it is far less sensitive than rods or cones to light intensity and thus requires much brighter light for its activation [1,4,6]. The melanopsin-mediated, or intrinsic, activation of ipRGCs is characterized by delayed onset (from appr.1 s) to firing, slow rise to maximal firing rate, high resistance to fatigue during continuous light stimulation and persistence of activity even after light termination, whereas outer photoreceptor responses to long wavelength light are characterized by a rapid firing decline during illumination with no recordable firing after light termination [1,4].

In in vivo human studies, the pupil responses to short wavelength (480 nm) light, in particular the post-illumination pupil response, has been shown to parallel the ipRGC firing pattern and thus can be used as a marker of melanopsin-driven ipRGC activity in vivo [6-14].

Because many physiological functions in the human body are characterized by rhythmic changes within an approximate 24-hour period generated by the endogenous circadian pacemaker, the importance of sufficient amounts of daytime light reaching the retina is gaining greater attention [15-20]. There is also growing evidence that disorders of the sleep-wake rhythm, alterations in mood, and poor performance on cognitive and behavioural tests in humans are, in part, related to an insufficient amount of exposure to environmental light [21-23]. Circadian misalignment has certainly been implicated in sleep disturbance, insomnia and depression, particularly in the elderly population in whom 40–70% suffer some form of chronic sleep disturbance.

As the human body ages, specific anatomical and physiological changes occur which could influence the amount of light that reaches retina. One such age-related change is steady-state pupil size. Many studies have reported that baseline pupil size (both dark and light adapted) decreases linearly with increasing age in healthy subjects after the pupils reach a peak baseline size during adolescence [24-28].

Another light reducing factor, strongly associated with biological aging, is structural changes of the lens that lead to reduced transmission of the visible short wavelength light and reduced retinal illumination. In contrast, the cornea, aqueous and vitreous humour transmits nearly all visible light [29,30]. In the human lens, formation of lens fiber cells is a continuous process. With age, the lens increases in size and accumulates lens proteins which undergo posttranslational modifications that change the optical characteristics of the lens, giving it a yellow or brown appearance when observed at the slitlamp. As lens proteins are autofluorescent when exposed to short wavelength (blue) light, the age related change in lens transmission is paralleled by increased autofluorescense. Thus, in vivo quantification of lens transmission is based on autofluorescence of lens proteins [31-33]. The yellow lens works as an effective color filter reducing a large portion of transmitted blue light. Approximately 1% of lens transmission is lost per year after the age of 18 years [33,34], and, thus the effect of blue light transmission through the lens measured in vivo might need to be a consideration in studies using quantification of pupil responses to blue light. However, recent studies with healthy subjects did not reveal age impact on melanopsin driven postillumination pupil response [10].

As a first step in the investigation of age as a possible modulating factor for the ipRGC activity level, we investigated the profile of melanopsin mediated pupil responses, as compared to pupil responses mediated by outer photoreceptor signals, in healthy adult subjects over a wide age range.

As a second step, we examined the relationship between an in vivo measured lens transmission of blue light and pupil responses to monochromatic blue light at various intensities in the healthy subjects in order to better understand, if the normal physiological changes in the aging lens might modulate certain light dependent responses of the eye, such as melanopsin activation.

## Methods

### Subjects

Forty-five healthy adult subjects (24 men, 21 women: age range 26–68 years, Mean (± SD) was 44 (± 14) years) participated in this study. Seven to ten subjects were included at every age decade to ensure an even distribution though the age range: 20–29 years (11 subjects), 30–39 (8), 40–49 (9), 50–59 (7), 60–69 (10).

Informed consent was obtained from all participants and the study was carried out in compliance with the Declaration of Helsinki. The study was approved by the local ethics committee.

None had any history of any systemic or ocular pathology or use of any medication that could influence the pupil light reflex. All subjects underwent a baseline ophthalmologic examination which included ETDRS visual acuity, Ishihara test for color blindness, Farnsworth D-15 color hue test, swinging flash light test for relative afferent pupillary defect, slit lamp biomicroscopy and fundoscopy without pupil dilation, and visual field testing (Humphrey, SITA standard 24–2 program, San Leandro, CA). Additionally, subjects underwent macular and optic disc scanning using optic coherence tomography (OCT4, Cirrus HD-OCT, Carl Zeiss Meditec, Inc., Dublin, CA) in order to rule out any anatomical changes in the macula or in the retinal nerve fiber layer. Lens transmission for blue light was measured with an ocular fluorometer (Fluorotron Master, OcuMetrics, Inc., Mountain View, CA) as an indicator of nuclear sclerosis and will be analyzed in a separate paper. All tests were within normal limits for 44 subjects, and one subject from the age group 20–29 years was excluded because of impaired color vision which was presumed congenital.

### Chromatic pupillometry, light stimulation and protocol

The pupil response to light of a non stimulated eye (consensual pupil response) was recorded by a prototype infrared monocular chromatic pupillometer (IdeaMedical, Copenhagen). A diffuse monochromatic light (narrow bandwidth LEDs, of either 470 nm with 20–22 nm full width at half maximum or 660 nm wavelength with 20–22 nm full width at half maximum) was presented to one undilated eye at a fixed distance. The timing of the light stimulus was computer controlled and is described below.

Data was smoothed using a nearest neighbour approach, i.e., each data point was compared to the next point and recalculated as the mean of the start point and the three adjacent points to each side. Blink artefacts were evaluated by the inbuilt algorithm and defined as a large change for 5 successive time points. If present, all data points within one second were substituted by a linear regression line calculated from the point immediately before the blink to the value at the next second. Data was exported and stored on a computer for later offline analysis. The pupillometer luminance output was calibrated initially by a spectrophotometer PR-655 (Photo Research, Chatsworth, CA, USA), and the luminance was re-checked before each session at the same distance and angle as the position of the patient’s cornea. This chromatic pupillometer, stimulus calibration, light safety issues and data processing details have been previously described [35].

Recent studies have indicated that pupil responses to lower intensity blue light are mediated predominantly by the outer photoreceptor (rod-cone) system, whereas responses to higher intensity blue light reflect input from the melanopsin activation in the ipRGCs [6,9] as well as contribution of the outer photoreceptors [9,36]. Light intensities of 13.6 log quanta/cm^2^/s and higher [6,13,14] have been shown to effectively activate melanopsin in vivo.

We performed 6 subsequent tests using red (660 nm) or blue (470 nm) light stimuli of different intensities in the following order: 300 cd/m^2^ red light (corresponding to 14.9 log photons/cm^2^/s), 300 cd/m^2^ blue light (corresponding to photon flux irradiance of 14.8 log photons/cm^2^/s) and 4 subsequent tests using stepwise increasing intensity of blue light stimulus: 3, 30, 100, and 300 cd/m^2^.

The red light of 300 cd/m^2^ intensity used in this study lies at the limit of the spectral sensitivity of melanopsin and would not be expected to strongly activate melanopsin, but would rather activate the M and L cones [4] and would also serve as a control stimulus to reflect non specific influences as fatigue on pupil light response.

Based on the scotopic, mesopic and photopic thresholds of the photoreceptors and previously reported data on chromatic pupillometry, a 3 cd/m^2^ blue light stimulus was chosen to reflect activity of rods and cones. Increasing photopic levels of 30, 100 and 300 cd/m^2^ increasingly recruit cone input as well as melanopsin. The 300 cd/m^2^ blue light condition used in this study was specifically selected to activate melanopsin preferentially over rods and cones, as melanopsin has peak sensitivity in the action spectrum of light around 480 nm and requirement of high intensity [1,4,6]. For this study, the 300 cd/m^2^ light stimulus is referred to as the high intensity stimulus.

In order to minimize possible circadian variations of the pupil light response as has been recently reported [13], all tests were performed during the day.

Each subject sat in a quiet room under mesopic (0.74 cd/m^2^) lighting conditions for four minutes. Then all light sources in the room were turned off, and the pupil was adapted to darkness (0 cd/m^2^) for one minute. Thereafter, three measurements of the dark adapted pupil diameter were obtained and converted to millimetres using a nomogram scale of artificial pupils.

The nonstimulated pupil was continuously recorded during the following light sequence: 10 seconds of prestimulus dark period, 20 seconds of a continuous light stimulation, then 1 minute of poststimulus dark period.

The test sequence and light intensities are described above. All tests were separated by an approximately 5 minute pause, while the subject remained in an upright position in the room under mesopic light.

### Main pupil outcome parameters

In this study, different aspects of the pupil response to continuous light stimulation were assessed in order to better understand the effect of different photoreceptor inputs on the recorded pupil waveform. The outcome parameters are described below.

#### Determination of a baseline pupil size and a normalized pupil size

For each test session, a baseline pupil size (BS), was calculated as a mean diameter within 10 seconds in dark before light stimulation. The pupil size was reported as a normalized pupil size (NPS), i.e., a ratio in which a measured actual pupil diameter is divided by BS.

#### Maximal pupil contraction amplitude (maximal CA)

This is the maximal difference (expressed as %) in NPS from BS within the first 6 seconds of light stimulation. This parameter is largely a measure of light evoked outer retinal photoreceptor (cones and rods) signalling on the ipRGCs [6,9,36,37].

#### Sustained pupil contraction amplitude (sustained CA)

This is the difference (expressed as %) in NPS from BS at the 20^th^ second after light onset, i.e., at the last second of continuous light stimulation. This parameter reflects the contribution not only from melanopsin activation and modulation of the pupil response by intrinsic ipRGC activity, but also from outer photoreceptors [9,36].

#### Poststimulus area under the curve (poststimulus AUC)

This outcome parameter is intended to assess the post-illumination pupil response in healthy human eyes [6,7,9,11-14] and to reflect the whole dynamics of the pupil redilation over time after light stimulus termination. Thus the post-illumination pupil response amplitudes were summed and reported as the *area under the curve (AUC)*[35]. The AUC is calculated as followed: AUC = ($AUC=\sum_{t0}^{t1}1.0-\text{NPS}$), where t_0_ is starting time point of pupil response summation, t_1_ is stopping time point of summation, 1.0 is a baseline pupil size (BS), and NPS is normalized pupil size (actual pupil size divided by BS).

**Figure 1:**
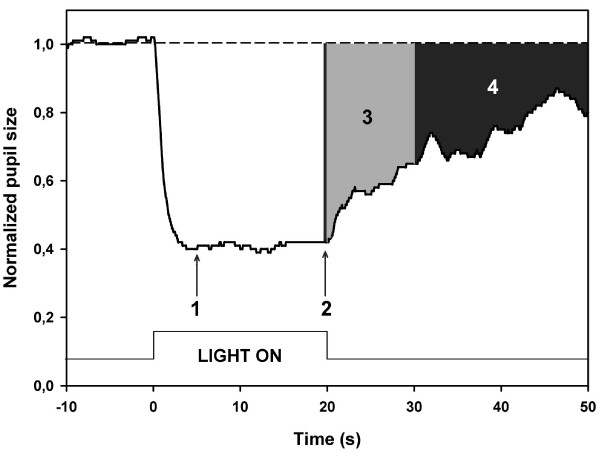
**Graphic representation of the pupil response to blue light in one normal subject.** The dotted line at the top is an extrapolation from baseline pupil size (BS) and corresponds to 1.0 on the Y-axis. Y-axis represents values of the normalized pupil size (NPS). Time −10 is the start of video recording of the pupil while in darkness.. Time 0 is the onset of a 300 cd/m^2^ continuous blue light stimulus having duration of 20 seconds (vertical bars at the bottom). The arrow at point 1 shows where the maximal contraction occurs (the maximal CA). The arrow at point 2 is the 20^th^ second of light stimulation and represents the sustained CA. The third parameter, 3, is the early poststimulus AUC (grey shaded area in the pupillogram) which is the summed pupil response during the first 10 s after light termination. The late poststimulus AUC, 4 (black shaded area), another summed post-illumination response parameter is taken from the 10^th^ to 30^th^ second after light termination. For definitions, see Methods.

Two poststimulus AUCs were calculated: AUC during early poststimulus phase (0–10 s after light offset) and AUC during late poststimulus phase (10–30 s after light offset). Figure 1 is an example of a waveform pupillogram on which these measured parameters are indicated.

### Lens transmission

Lens transmission was measured in a study eye of each subject by ocular fluorometer (Fluorotron Master, OcuMetrics, Inc., Mountain View, CA) with blue excitation light ranging from 430–490 nm (peak at 480 nm) and green emission light ranging from 530–630 nm. Three consecutive measurements were taken for each subject, and mean transmission was calculated. The theoretical maximal transmission of 1.0 indicated a totally clear lens.

**Figure 2:**
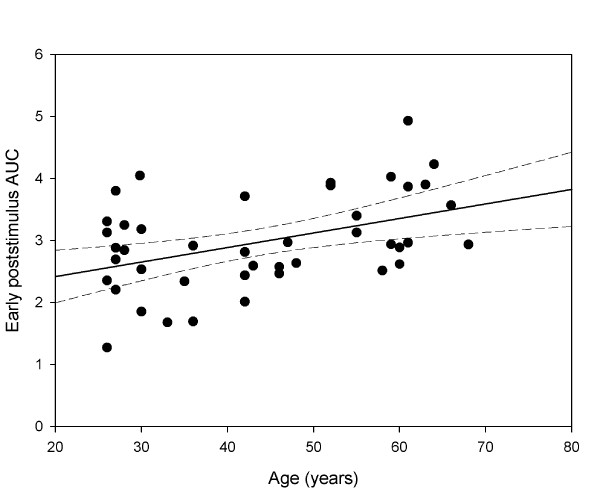
**Lens autofluorescence scan.** Lens autofluorescence of a 28 year old subject (**A**) and a 61 year old subject (**B**). The fluorescence intensity is given as ng/ml. The x-axis represents a visual axis in mm. Ppost is a peak fluorescence of the lens posterior pole, and Pant is a peak fluorescence of the lens anterior pole. In the older subject values of the peaks appear higher and the difference between posterior and anterior peaks is greater than that of the young subject. Due to the accumulation of denaturated lens proteins in the advanced age lens, light absorption increases, and the back emitted fluorescent light from the posterior peak appears less intense than the light from the anterior peak.

An example of in vivo measured amounts of fluorescent light (from the posterior pole and from the anterior pole) in a young subject and in an advanced age subject is shown in Figures 2A,B. In the ageing lens, accumulation of denaturated lens proteins causes increased light absorption, especially of short wavelength (blue) light, and, thus the back reflected fluorescent light from the posterior peak decreases.

Lens transmission was calculated as the ratio between the amount of fluorescent light emitted from the lens posterior pole and the amount of light emitted from the anterior pole. As the observed transmission may be biased due to differences in the light absorption and the fluorescence at the same wavelength, lens transmission was corrected based on an in vitro validation of fluorophotometric transmission measurements on human donor lenses [33].

### Statistical analysis

Statistical Analysis System (SAS) software (SAS version 9.1, SAS Institute Inc., Cary, NC, USA) was used for statistical analysis. A p-value < 0.05 was considered significant for all tests and two sided tests were used.

Data were analysed with a general linear model adjusted for baseline pupil and gender. As described under Methods, the pupil size is calculated relative to the baseline pupil, but a residual influence was observed in the linear model and gender and baseline pupil diameter was retained as covariates.

The outcome parameters approximated a normal distribution with homogenous variance and residual plots were performed for all type of analysis. Variance is known to increase for late post-illumination response [35], and, therefore, results for post-illumination pupil response parameters were also analysed by non-parametric tests. The non-parametric tests confirmed the conclusions obtained by parametric models.

Linear correlation analysis was applied for age vs. dark-adapted baseline pupil as well as age vs. lens transmission and reported as Pearson correlations. A paired t-test was used for comparison of outcome parameters between the first and second test at 300 cd/m^2^ blue (470 nm) light conditions, as well as between red (660 nm) and blue (470 nm) high intensity (300 cd/m^2^) tests.

## Results

### Age and pupil

#### Pupil response parameters to high intensity 300 cd/m^2^ blue (470 nm) stimulus in relation to age

**Figure 3:**
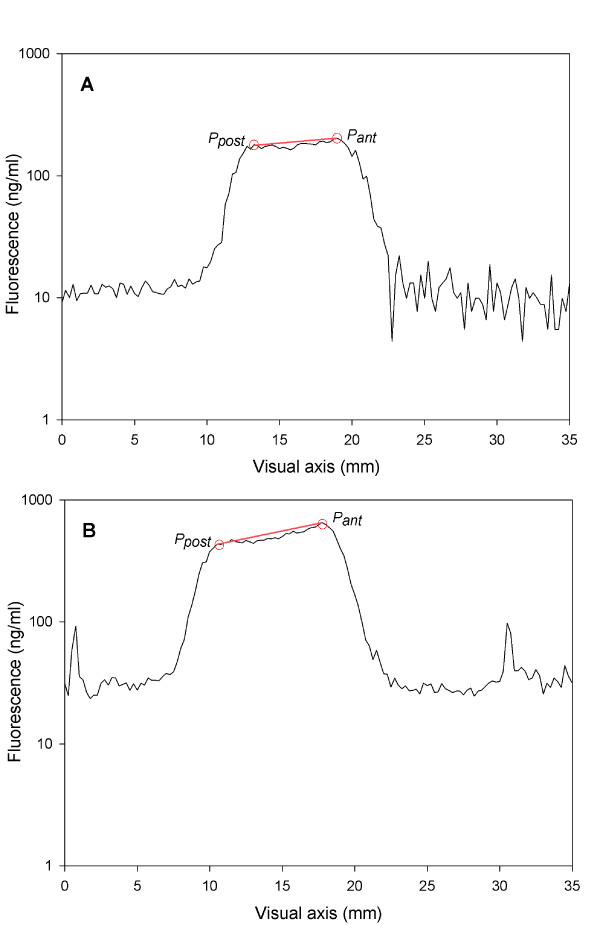
**The correlation between age and early poststimulus pupil response (AUC) at 300 cd/m**^**2**^**blue light conditions.** The linear correlation of age to the summed poststimulus pupil response within the first 10 seconds after light termination (early AUC 0–10) was highly significant (Pearson correlation coefficient r = 0.43), with increasing age corresponding to a larger pupil contraction (solid line is regression line, the dashed lines represent the 95% CI).

The maximal CA to 300 cd/m^2^ blue light did not change with age (*p* = 0.61), however, at this high intensity, the sustained CA was positively correlated to increasing age (*p* = 0.02, Table 1). The early but not late poststimulus AUC correlated significantly to age (*p* = 0.0014, Table 1 and Figure 3) for high intensity blue light, although the trend of increasing AUC with age was observed for late AUC as well.

**Table 1:** **Pupil responses to blue light and red light of equiluminant intensity (300 cd/m**^**2**^**) and their correlations to age**

	***Test condition***				***Correlation to age***		
***Para-Meter***	***Light color***	***Light, intensity cd/m***^***2***^	***Mean***	***95% CI***	***b***	***R***^***2***^	***p value***
**LIGHT ON PARAMETERS**							
**Maximal CA, %**	red	300	43.5	41.1–46.0	–0.008	0.06	0.95
	blue	300	53.7	51.5–55.9	0.05	0.02	0.60
**Sustained CA, %**	red	300	37.2	34.5–40.0	0.18	0.09	0.16
	blue	300	52.4	50.1–54.7	0.23	0.14	0.02*
**LIGHT OFF PARAMETERS**							
**Early post-stim. AUC**	red	300	1.6	1.4–1.7	–0.0002	0.04	0.97
	blue	300	3.0	2.7–3.2	0.03	0.25	0.0014*
**Late post-stim. AUC**	red	300	0.7	0.6–0.9	–0.01	0.008	0.15
	blue	300	2.6	2.0–3.2	0.05	0.11	0.09

Pupil response parameters are given as mean with 95% confidence interval.

The correlation between pupil responses and age was calculated by a multiple linear regression with adjustment for baseline pupil size and gender. Results are given as: b, the slope of the regression (response change per year), R^2^, the correlation coefficient, i.e., the fraction of variance explained by the regression model and the p-value.

*Contraction amplitudes (CA):* Maximal CA is difference between baseline pupil size (BS) and the normalized pupil size (NPS) at maximal contraction to light. Sustained CA is the difference between BS and NPS during the last second of light stimulation.

*Areas under the curve (AUC):* Early poststimulus AUC is the summed response amplitudes within 0–10 s after light termination. Late poststimulus AUC is the summed response within 10–30 s after light termination.

Light stimulus luminance of 300 cd/m^2^ red (660 nm) or 300 cd/m^2^ blue (470 nm) light corresponded to 14.9 and 14.8 log photons/cm^2^/s, respectively.

* Level of significance p < 0.05. CI, confidence interval; b, slope of the regression; R^2^, correlation coefficient; CA, contraction amplitude; AUC, area under the curve (the summed pupil response amplitudes within the given time window.

In order to evaluate for possible influence of a rod/cone effect in terms of rapid re-dilation (lasting appr.5 s after light offset) on the early poststimulus AUC (AUC 0–10 s after light offset), we additionally calculated the poststimulus AUC within the 5^th^ and 15^th^ second after light stimulus termination. There was no significant difference between this parameter (AUC 5–15) and the early AUC (0–10 s) at high intensity blue light conditions, and similarly, the AUC 5–15 did show significant increase with age by 0.03 (b) per year of age (*p* = 0.008) with correlation coefficient R^2^ of 0.20 explaining an age impact of 20% on this AUC increase.

#### Pupil response parameters in relation to age at 300 cd/m^2^ red (660 nm) stimulus conditions

In contrast to the pupil response parameters to high intensity blue light, none of the pupil response parameters (maximal CA, sustained CA, early and late AUC) to high intensity red light showed a significant correlation with age, and no interactions were found, indicating that the observed age related increase in the sustained CA and early poststimulus AUC to blue light were wavelength specific findings.

#### Comparison of pupil response to blue versus red light stimulus of high intensity (300 cd/m^2^)

All pupil response parameters (maximal CA, sustained CA, early and late AUC) to blue light were significantly larger than those to red light (*p* < 0.0001).

#### Comparison of pupil response to high intensity blue light obtained in the beginning and the end of the experiment

The pupil response comparison analysis between the first-presented and second-presented high intensity blue light stimulus did not reveal a significant difference for any of the response parameters (maximal CA, sustained CA, early and late AUC), indicating that in this study, there was no influence of the preceding light stimulus (300 cd/m^2^ red vs. 100 cd/m^2^ blue) on the subsequent 300 cd/m^2^ blue light stimulus.

#### Dark adapted baseline pupil size (BS) in relation to age

The BS ranged from 6.9 mm to 8.7 mm and decreased with increasing age by approximately 0.05 mm per year of age (r = 0.62, *p* < 0.0001). There was no effect of gender on the dark-adapted BS.

### Lens transmission and pupil responses to blue (470 nm) light

#### Lens transmission of blue light in relation to age

**Figure 4:**
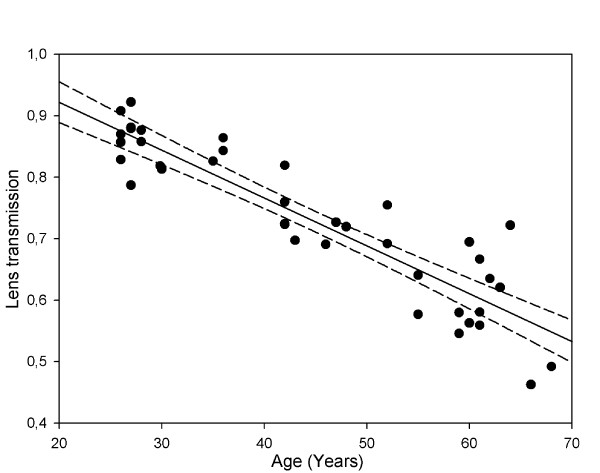
**The relation between age and lens transmission of blue light.** Lens transmission correlated negatively to age, with lens transmission decreasing significantly with increasing age. The rate of change was approximately 1% per year of age (Pearson coefficient r = −0.91). Lens transmission of 1.0 corresponds to a totally clear lens in a young healthy subject (solid line is regression line, the dashed lines represent the 95% CI).

Our results revealed a highly significant linear decrease in blue (a peak at 480 nm) light transmission through the lens with increasing age (r = −0.91, *p* < 0.0001, transmission = 1.08–(0.01 * age), see Figure 4). Based on our data, the calculated blue light lens transmission in a 30 year old subject was 0.78, and in a 60 year old subject it decreased to 0.48.

#### Pupil responses in relation to stimulus intensity

**Figure 5:**
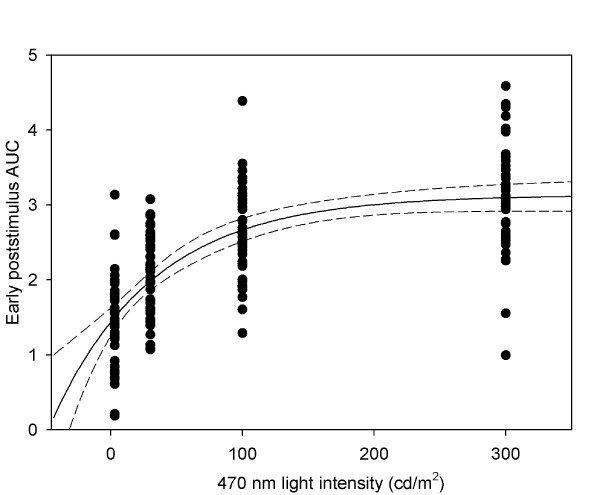
**The relation between early poststimulus pupil response and blue light intensity.** Early poststimulus response (AUC) versus 470 nm light intensity (cd/m^2^). The pupil response increases as intensity increases, but levels off at intensities approaching 300 cd/m^2^ (solid line represents an exponential fitted curve, the dashed lines is 95% CI).

All pupil response parameters, i.e., maximal CA, sustained CA, poststimulus early AUC and poststimulus late AUC, increased significantly with increasing light intensity. For relation to the nominal intensity, the pupil response increased nonlinearly and indicated a saturation phenomenon between 100 and 300 cd/m^2^ (Figure 5A), whereas the relation between log transformed light intensity (in the range of 3–300 cd/m^2^) and pupil response was linear (*p* < 0.0001 for all parameters): per 1 log unit of light intensity, maximal CA increased by 6.8% (R^2^ = 0.26), sustained CA increased by 10% (a correlation coefficient R^2^ = 0.44), early poststimulus AUC increased by 0.8 (R^2^ = 0.50) and late poststimulus AUC increased by 0.9 (R^2^ = 0.24). Thus, the strongest log linear correlation of the response to increasing stimulus intensity was found for the early poststimulus AUC.

#### Pupil responses in relation to lens transmission and stimulus intensity

**Figure 6:**
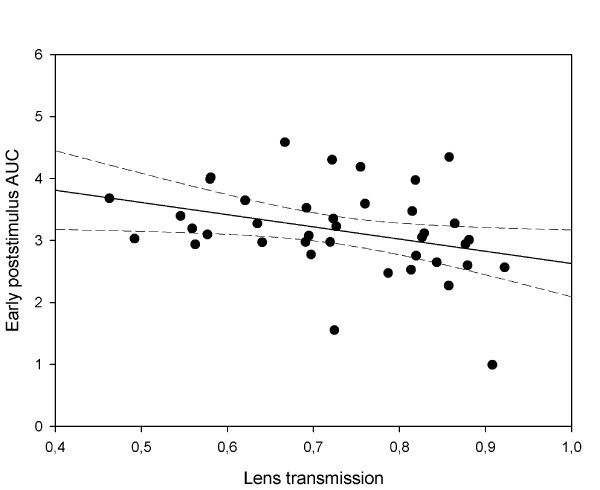
**The relation between early poststimulus pupil response at 300 cd/m**^**2**^**and lens transmission of blue light.** The early poststimulus pupil response (AUC) increased significantly when lens transmission decreased (Pearson correlation coefficient r = − 0.38), i.e., the greater poststimulus pupil response was observed in advanced age subjects with denser lenses (solid line is regression line, the dashed lines represent the 95% CI).

A negative correlation was found between pupil responses and lens transmission, i.e., in young subjects with relatively clear lenses pupils responded less to light, and, oppositely, in older subjects with denser lenses pupils responded greater, despite the significant loss of blue light transmission through the lens with age (Table 2 and Figure 6). The tendency to a negative correlation between pupil response and lens transmission was only significant for early poststimulus AUC at 300 cd/m^2^ light intensity with the correlation coefficient R^2^ value of 0.18 (p < 0.02), indicating a relatively low impact of lens transmission (18%) on the pupil response (Table 2, Figure 6). For the sustained response, a p-value of 0.06 indicated a similar effect, though it did not reach statistical significance.

**Table 2:** Pupil responses to blue (470 nm) light and their correlations to lens transmission

*Parameter*	*Stimulus intensity cd/m*^2^	*Mean *	95% CI	***Correlationto age***			***Correlationto lens transmission***		
				*b*	*R*^*2*^	*p value*	*b*	*R*^*2*^	*p value*
**LIGHT ON PARAMETERS**									
**Maximal CA, %**	3	41.7	38.2–45.1	0.21	0.09	0.18	–0.17	0.08	0.34
	30	48.4	46.0–50.9	0.19	0.07	0.10	–0.15	0.06	0.23
	100	51.8	49.5–54.0	0.10	0.04	0.33	–0.02	0.01	0.89
	300	55.3	53.4–57.3	0.16	0.09	0.07	–0.07	0.05	0.44
**Sustained CA, %**	3	33.3	29.9–36.7	0.30	0.14	0.048*	–0.21	0.09	0.22
	30	44.6	42.4–46.9	0.17	0.10	0.09	–0.13	0.07	0.25
	100	49.7	47.6–51.8	0.19	0.12	0.043*	–0.12	0.06	0.22
	300	52.5	50.4–54.5	0.23	0.15	0.014*	–0.20	0.11	0.06
**LIGHT OFF PARAMETERS**									
**Early post-stim. AUC**	1.5	1.3–1.7	0.02	0.15	0.016*	–0.01	0.06	0.21	
	30	2.1	1.9–2.2	0.01	0.08	0.09	–0.004	0.02	0.63
	100	2.6	2.4–2.8	0.02	0.19	0.005*	–0.01	0.08	0.22
	300	3.1	2.9–3.3	0.03	0.29	0.0003*	–0.03	0.18	0.02*
**Late post-stim. AUC**	3	0.8	0.6–1.0	0.01	0.05	0.20	–0.002	0.02	0.88
	30	1.2	0.9–1.5	0.008	0.04	0.56	0.009	0.04	0.60
	100	1.8	1.4–2.3	0.02	0.05	0.30	0.009	0.06	0.69
	300	2.8	2.4–3.3	0.04	0.08	0.07	–0.03	0.04	0.29

Each pupil response parameter is given as mean and its 95% confidence interval.

Correlations between pupil responses and age and between responses and lens transmission were calculated with adjustment for baseline pupil size and gender using a multiple linear regression model. Results are given as: b, the slope of the regression (response change per transmission unit), R^2^, the correlation coefficient, i.e., the fraction of variance explained by the regression model and the p-value.

For abbreviations and definitions of parameters, see Table 1 and Methods.

Blue (470 nm) light stimulus luminance of 300 cd/m^2^ corresponded to photon flux irradiance of 14.8 log photons/cm^2^/s.

* Level of significance p < 0.05. CI, confidence interval; b, slope of the regression; R^2^, correlation coefficient; CA, contraction amplitude; AUC, area under the curve.

*Substitution of a lens transmission variable by age variable in correlation analysis* revealed that the significant effect of age was larger than that of lens transmission for sustained pupil response and early poststimulus AUC at 300 cd/m^2^ blue light: R^2^ of 0.15 (*p* = 0.014) and 0.29 (*p* = 0.0003), respectively. For intensities below 300 cd/m^2^, significant correlations to age were also found for responses at 3 and 100 cd/m^2^ light conditions (Table 2).

#### Comparison of lens transmission and pupil responses in a 30 year old subject versus a 60 year old subject

The theoretically calculated values of lens transmission and pupil responses during exposure to light and after light termination for a subject aged 30 years versus a subject aged 60 years are shown in Table 3. Even though the blue light transmission decreased by almost 40% from 30 to 60 years, the pupil constriction to blue light increased, particularly for the sustained response and the early poststimulus response by appr.10% and 20%, respectively. In other words, lens related loss of blue light intensity and its subsequent reduction in retinal illumination did not lead to a decreased pupil response to blue light.

**Table 3:** Comparison of parameter change with age in a 30 year old subject versus a 60 year old subject

**Parameter**	**Intensity cd/m**^**2**^	**30 yrs.**	**60 yrs.**	**%Change with age**
Lens transmission		0.78	0.48↓	–38
Baseline pupil, mm		8.20	6.70↓	–18
Max. CA, %	300	53	56↑	4
Sust. CA, %	300	51	54↑	7
AUC early post.	300	2.6	3.1↑	17

CA, contraction amplitude; AUC, area under the curve.

*Lens transmission.* As age doubles, blue light transmission decreases by appr.40% and dark adapted baseline pupil diameter decreases by appr.20%. *Pupil response parameters.* An increased response was observed with increased age especially for early poststimulus AUC.

For abbreviations and definitions of parameters, see Table 1 and Methods.

In contrast, an increase in stimulus intensity from 3 cd/m^2^ to 300 cd/m^2^ (by 2 log units) during the period of the experiment led to a marked and significant increase in pupil response both during exposure to light and after light termination in a log linear fashion: sustained CA increased by appr.40% per 2 log units and early poststimulus AUC increased by appr.50% per 2 log units in a young subject and an older subject.

## Discussion

We have examined the effects of the age on the pupil response to high intensity (300 cd/m^2^) long (660 nm, red) or short (470 nm, blue) wavelength light.

Pupil responses to red light did not correlate to age. As both the baseline pupil size and the lens transmission of blue light decreases with age, a reduction in pupil response to blue light might be expected. We found a significantly enhanced response for sustained and early poststimulus pupil contraction at high intensity blue light stimulus condition.

For the maximal pupil contraction and the late poststimulus response at either red or blue high intensity light conditions, the effect of age was non-significant with a small numerical increase. The lack of a correlation between age and the maximal pupil contraction amplitude suggests that the outer photoreceptors (rods and cones) input to the neural signal of the ipRGCs to the pupil light reflex is relatively resistant to change due to the aging process. For the late poststimulus pupil response (assessed as the sum of amplitudes (area under the curve, AUC) within 10 to 30 s after light offset), a possible reason for the lack of correlation to age could be increasing contribution of supranuclear influences on the pupil size which results in fluctuating pupil size during the late phase of re-dilatation and, thus, greater variability [35].

In contrast, advancing age did positively correlate with increasing magnitude of the sustained pupil contraction during continuous light stimulation, as well as the early post-illumination pupil response (poststimulus AUC within 0 to 10 seconds after light offset), but this relationship was specific to the short wavelength light stimulus condition.

The enhanced response (sustained and early poststimulus contraction) to high intensity blue light was found, despite the known decrease in lens transmission (in our study found to be 1% per year) and adjustment of outcome results for the baseline pupil. If age was substituted by the in vivo measured lens transmission of blue light in the correlation analysis, the pupil response change to blue light was only found significant for the early poststimulus AUC at high intensity stimulus conditions. The lower correlation between the pupil response and the lens transmission, as opposed to age, indicates that the effect of transmission cannot, by itself, account of the effect of age on the early poststimulus AUC, presumably a marker of the melanopsin-mediated ipRGC activity.

The finding that the pupil maintains a contracted state during and after continuous blue light stimulation of high intensity is not unique to our study and, in fact, has been demonstrated to be the signature feature of melanopsin activation [6,7,9-14].

The novel finding of our study is that these physiological markers of melanopsin-mediated ipRGC activity become more robust with increasing age. This is rather an unexpected finding, given that certain physiologic changes in the eye that occur with age, such as yellowing of the lens and attrition of retinal ganglion cells [38], might intuitively be expected to cause decreased pupil responses to blue light with increasing age.

This suggests that there exist other age related factors that might work to enhance the melanopsin-mediated pupil responses. One of the factors could be the light scatter of the lens which is known to increase with age in parallel to the decrease in lens transmission [39-41]. The scatter leads to a changed distribution of light over the retina, and one might speculate, if this could increase activation of the unevenly distributed melanopsin containing ipRCGs.

Could it be that ipRGCs undergo some age related adaptive processes in order to keep their functioning at an optimal level? Some evidence exists that structural changes appear in rod outer segments, i.e., they increase with age giving a greater functional capacity, while the number of rods decreases with age [42].

There is also some data on age-related changes that occur in the suprachiasmatic nucleus (SCN) in terms of unchanged neuron number with increasing age, which might lend support to the notion that the optic pretectal nucleus (OPN) (the nucleus which integrates the various afferent signals driving the pupil light reflex) might undergo similar changes as well [43].

The additional finding of a log linear increase in pupil response with increasing light intensity has been demonstrated previously in experimental studies [4,6] and, in this study, did not show any threshold effect within the intensity range of 3 to 300 cd/m^2^.

Our example where we compared pupil responses of a 30 year old subject to a 60 year old subject supports the adaptation hypothesis: a gradual and great decrease in blue light transmission with years resulted in enhanced pupil responses (sustained and early poststimulus contraction) to bright blue light by appr.10–20%, whereas a decrease in light intensity by 2 log units (from 300 to 3 cd/m^2^) during the experimental procedure led to a linear decrease in these pupil responses to blue light by 30–50% independently of age.

It is beyond the scope of this study to understand where in the pupil light reflex pathway that this augmentation of pupil response to blue light seen in older healthy subjects occurs. Additional studies are needed to clarify which other mechanisms are maybe responsible for enhancement of these ipRGC mediated pupil responses occurring with age.

## Conclusions

In conclusion, advancing age does not reduce, but rather increases the effectiveness of bright blue light to activate ipRGCs, as determined from pupil responses. This effect of age does not appear to be due simply to reduced retinal illumination from the natural age-related decrease in lens transmission of blue light. Thus, other age-related factors must play a role in the enhancement of the intrinsic ipRGC activity that occurs with advancing age.

## Competing interests

Aki Kawasaki discloses that she has received a personal compensation from Bayer SpG for panel advisory work in an unrelated topic in the past. The authors declare that they have no competing interests.

## Authors’ contributions

AK and KH contributed to the study design and interpretation of data, drafting and revising the manuscript. HLA contributed to the study design and to revising the manuscript. BS contributed to the study design, statistical analysis and to revising the manuscript. MSH and AEB contributed to acquisition of data and to drafting the manuscript. LK contributed to interpretation of data and to critical revision of the manuscript. All authors have given the approval to the final version.

## Pre-publication history

The pre-publication history for this paper can be accessed here:

http://www.biomedcentral.com/1471-2415/12/4/prepub
